# Serologic Evidence of Dengue Infection before Onset of Epidemic, Bangladesh

**DOI:** 10.3201/eid0911.030117

**Published:** 2003-11

**Authors:** M. Anowar Hossain, Mahmuda Khatun, Farzana Arjumand, Ananda Nisaluk, Robert F. Breiman

**Affiliations:** * ICDDR,B: Centre for Health and Population Research, Mohakhali, Dhaka, Bangladesh; † Armed Forces Research Institute of Medical Science, Bangkok, Thailand

**Keywords:** seroprevalence, dengue, typhoid, Widal, Japanese encephalitis, MAC ELISA

## Abstract

Dengue fever emerged in Bangladesh in 2000. We tested 225 serum and plasma samples from febrile patients and 184 blood donors in 1996 and 1997 for dengue antibodies; 55 (24.4%) febrile patients had dengue antibodies (65.5% with secondary infection pattern), compared with one (0.54%) donor (p < 0.001), suggesting that dengue transmission was ongoing well before 1996.

Dengue continues to spread globally; two fifths of the global population is at risk, primarily within tropical countries (*1*,*2*). A proportion of dengue infections result in dengue hemorrhagic fever (DHF), which is associated with high death rates. Most deaths are preventable with timely, careful fluid management. In areas hyperendemic for dengue with clinicians experienced in diagnosis and management of dengue fever and DHF, death rates are relatively low (*3*,*4*). Recognition of ongoing dengue transmission is helpful for optimal management and implementation of rational prevention programs (*5*).

While dengue viruses were likely responsible for what was called Dhaka fever in 1965 (*6*), dengue fever and DHF were not recognized in recent decades in Bangladesh, until an outbreak occurred in 2000 (*7*). Nearly 15,000 patients have been hospitalized in Dhaka and other urban areas in Bangladesh since 2000. News reports focus daily on the numbers of new dengue cases, and panic is palpable among residents of Dhaka. We assisted the Government of Bangladesh in designing and implementing emergency strategies to contain the epidemic. Serologic responses of dengue patients (based on evaluating immunoglobulin [Ig] M/IgG ratios) identified during surveillance showed that approximately 70% of patients had also been infected with dengue previously (*7*), suggesting that unrecognized dengue illnesses had been present.

The objective of this study was to evaluate whether undiagnosed dengue infection was occurring in Bangladesh before 2000. We studied stored serum samples from a group of febrile patients who attended the Clinical Laboratory of ICDDR,B during 1996 and 1997 and who were evaluated for typhoid.

## Materials and Methods

Acute-phase serum specimens, which had been submitted for Widal testing for evaluation of typhoid fever, were identified from 225 febrile patients who attended the Clinical Laboratory of ICDDR,B during 1996 and 1997; specimens were stored at –20°C. We also identified serum samples from 184 blood donors obtained during the same interval and stored under similar conditions. Information about age and sex were not available for blood donors. All 409 serum specimens were tested for antibodies to dengue viruses and Japanese encephalitis virus (JEV) by IgM and IgG antibody–capture enzyme linked immunoassay (*8*,*9*).

Microtiter plates were coated with 100 μL goat antihuman IgM and IgG antibodies and incubated at 4°C for 48 to 72 h. Four coated plates were kept at room temperature for half an hour and washed five times with PBS-T (phosphate-buffered saline); 50 μL of diluted patient serum samples and positive and negative controls (1:100) were added into respective wells and incubated at 4°C overnight in a moisture box. After the plates were washed five times with PBS-T, 50 μL pooled antigens of dengue virus (DENV)-1–4 and 50 μL JEV were each added to separate wells and incubated at room temperature for 2 h. After the plates were washed five additional times to remove excess antigens with PBS-T, 25 μL working conjugate was added to each well and incubated at 37°C for 1 h. After the plates were washed with PBS-T five times to remove excess conjugate and PBS x 10 twice, 100 μL ortho-phenylenediamine (OPD) solution was added to each well and incubated at room temperature for 30 min. Finally, 50 μL stop solution (1 M sulfuric acid) was added to each well. An enzyme-linked immunosorbent assay reader measured the optical density (OD) at 492 nm.

OD values were used to calculate binding index and units of IgM and IgG. Binding index was defined as OD of test sample minus OD of negative control divided by OD of weak positive control (defined as 100 U) minus OD of the negative control. We multiplied binding index by 100 to obtain units of respective antibodies. Borderline results (±5 of 40 U) were repeated for validation.

IgM and IgG antibody values of >40 U were considered positive for dengue or JEV. When anti-dengue IgM or IgG values were >40 U, primary infection (first-time exposure) was defined as a ratio of IgM to IgG >1.8 and secondary infection (>1 previous exposure) was defined as a ratio of <1.8 (*10*).

Widal test was performed by rapid slide titration technique, as described by the manufacturer (Murex Biotech Ltd, Dartford, UK). A single test was performed on all acute-phase serum. A test was defined as positive when the titer was >1:80. However, recognizing the nonspecificity of this break point, we also considered titers of >1:320 or fourfold rise in serum antibody between acute- and convalescent-phase serum samples as representing “likely typhoid.” (*11*).

## Results

Among 225 febrile patients, 123 (54.7%) were male (Table 1). More than half (52.9%) of the patients were <15 years of age; most of the other patients were young adults <30 years old (32.9%).

**Table 1:** Age and sex distribution of 225 serum samples of febrile patients who attended the clinical laboratory of ICDDR,B, 1996–1997

Age group	Male, no. (%)	Female, no. (%)	Total
<5	21 (17.1)	19 (18.6)	40 (17.8)
5–15	46 (37.4)	33 (32.4)	79 (35.1)
16–29	38 (30.9)	36 (35.3)	74 (32.9)
>30	18 (14.6)	14(13.7)	32 (14.2)
Total	123 (54.7)^a^	102 (45.3)^a^	225 (100)

^a^Totals represent row percentages; all others are column percentages.

Fifty-five (24.4%) febrile patients had dengue antibodies, including 9 with antibodies reacting with JEV antigens; no dengue-negative serum samples reacted with JEV, suggesting that JEV antibody responses represented flavivirus cross-reaction (ratio of anti-dengue IgM units to anti-Japanese encephalitis IgM units were >1.0 in all 9) (9). In contrast, among 184 blood donors, one (0.54%) had measurable dengue antibodies (p < 0.001 when compared with febrile patients); none had JEV antibodies.

The male (22%) to female (27.5%) proportion of those positive for dengue antibodies was similar (Table 2). Among the 55 febrile patients with evidence of dengue infection, 36 (65.5%) had secondary antibody patterns, and 19 (34.5%) patients had primary patterns. Among those with dengue, secondary pattern was more common in female persons (75%) than in male persons (55.6%; p = 0.1). While not statistically significant, children <15 years old were more likely to have a primary pattern (12 [44.4%] of 27) when compared with people >15 years old (7 [25%] of 28).

**Table 2:** Distribution of patients positive for dengue primary or secondary antibody response by age and sex

Age group	Positive for dengue, no. (%)^a^				Total
	Male; n=27		Female; n=28		
	Primary	Secondary	Primary	Secondary	
<5	1 (33)	2 (67)	0	2(100)	05
5–15	8 (67)	4 (33)	3 (30)	7(70)	22
16–29	2 (33)	4 (67)	2 (18)	9(82)	17
>30	1 (17)	5 (83)	2 (40)	3(60)	11
Total	12	15	7	21	55

^a^Totals represent row percentages.

Widal test results were positive (1:80 dilution) in 52 (23.1%) serum samples from febrile patients. Widal test results did not correlate (negatively or positively) with dengue test results; 15 (28.8%) of 52 Widal-positive serum samples had evidence of dengue antibodies compared with 40 (23.1%) of 173 Widal-negative serum samples. When a stricter definition (>1:320 dilution) for a positive Widal test result was used, 3 (16.7%) of 18 positive serum samples had dengue antibodies compared with 52 (25.1%) of 207 negative serum samples (p > 0.5).

A substantial proportion (47.1%) of febrile patients were seen during July and August (Figure). Among 169 febrile patients during the rainy season and brief postrainy season (May-November, which mirrored the dengue season in Bangladesh during the years 2000 and 2001), 49 (29%) were positive for dengue compared with 6 (10.7%) of 56 febrile patients who were ill during December to April (p < 0.01).

**Figure:**
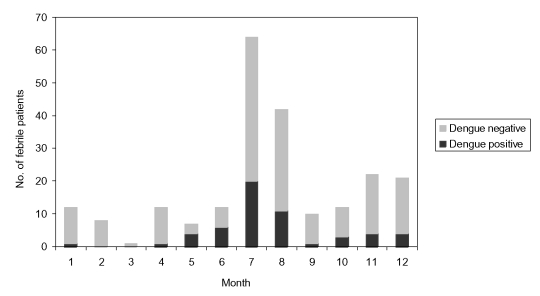
Distribution of results of dengue serologic testing by months.

## Discussion

Except for an epidemic in 1965 (*6*) and some isolated subsequent reports (*12*,*13*), dengue infection was not recognized as an important cause of illness in Bangladesh until 2000. The finding that febrile patients, but not blood donors from Dhaka from the same period, had dengue antibodies suggests that many of the febrile illnesses we evaluated in 1996 were caused by dengue, 4 years before the epidemic dengue was documented. Furthermore, most patients with dengue infection had antibody patterns consistent with previous infection, suggesting that dengue transmission had been ongoing well before 1996. Dengue illness was unrecognized most likely because it often is a self-limited influenzalike illness; more severe forms of dengue are confused with other illnesses prevalent in this tropical, impoverished, and densely populated, developing country.

While febrile patients described in this report were being evaluated for typhoid fever, it appears that they were actually more likely to have dengue. This finding underscores a need for access to diagnostic assays to confirm or broaden clinical suspicion. Diseases like dengue, typhoid, leptospirosis, and influenza, among others, may have signs and symptoms that are clinically indistinguishable. In some circumstances, laboratory confirmation can influence management and clinical outcome for the patient, as well as implementation of public health measures for prevention and control.

The Widal test is an imperfect test for typhoid, though specificity improves somewhat with rising titers (*14*). We did not observe such increases in specificity for typhoid, based on the proportion of patients with various Widal titers who had dengue antibodies. Some febrile patients may have had nonspecific stimulation of antibodies to O-antigens of enteric bacterial commensals resulting in false positive Widal tests (*11*). However, the possibility of concomitant infection caused by dengue and typhoid cannot be ruled out for some of these patients, given the exceedingly high incidence of typhoid in this region (*15*).

A widely held contention is that preexisting antibodies to dengue following previous exposure to the virus may predispose patients to more severe dengue illnesses, such as DHF and dengue shock syndrome because of antibody-dependent enhancement (*1*). In Bangladesh, thus far, only dengue serotypes 2 and 3 have been identified (*7*). Our findings suggest that, despite recent recognition of dengue illnesses in Bangladesh, previous exposure is not uncommon. We cannot be certain that DHF was also prevalent well before 2000, since comprehensive medical records needed for retrospective case identification are not available. However, if we assume that the antibody-dependent enhancement-risk hypothesis is correct, earlier dengue transmission within Bangladesh may be responsible for DHF cases now being observed and perhaps represents a substantial risk for greater incidence of DHF in the future, if new dengue serotypes are introduced.
